# Quasi‐Shell‐Growth Strategy Achieves Stable and Efficient Green InP Quantum Dot Light‐Emitting Diodes

**DOI:** 10.1002/advs.202200959

**Published:** 2022-05-26

**Authors:** Qianqian Wu, Fan Cao, Sheng Wang, Yimin Wang, Zhongjiang Sun, Jingwen Feng, Yang Liu, Lin Wang, Qiang Cao, Yunguo Li, Bin Wei, Wai‐Yeung Wong, Xuyong Yang

**Affiliations:** ^1^ Key Laboratory of Advanced Display and System Applications of Ministry of Education Shanghai University 149 Yanchang Road Shanghai 200072 P. R. China; ^2^ BOE Technology Group Co., Ltd. Beijing 100176 P. R. China; ^3^ The Institute of Technological Sciences Wuhan University Wuhan 430072 P. R. China; ^4^ CAS Key Laboratory of Crust‐Mantle Materials and Environments School of Earth and Space Sciences University of Science and Technology of China Hefei 230026 P. R. China; ^5^ Department of Applied Biology and Chemical Technology The Hong Kong Polytechnic University Hung Hom Kowloon Hong Kong 999077 P. R. China

**Keywords:** defect passivation, Indium phosphide, light‐emitting diodes, quantum dots, quasi‐shell

## Abstract

Indium phosphide (InP) based quantum dots (QDs) have been known as an ideal alternative to heavy metals including QDs light emitters, such as cadmium selenium (CdSe) QDs, and show great promise in the next‐generation solid‐state lighting and displays. However, the electroluminescence performance of green InP QDs is still inferior to their red counterparts, due to the higher density of surface defects and the wider particle size distribution. Here, a quasi‐shell‐growth strategy for the growth of highly luminescent green InP/ZnSe/ZnS QDs is reported, in which the zinc and selenium monomers are added at the initial nucleation of InP stage to adsorb on the surface of InP cores that create a quasi‐ZnSe shell rather than a bulk ZnSe shell. The quasi‐ZnSe shell reduces the surface defects of InP core by passivating In‐terminated vacancies, and suppresses the Ostwald ripening of InP core at high temperatures, leading to a photoluminescence quantum yield of 91% with a narrow emission linewidth of 36 nm for the synthesized InP/ZnSe/ZnS QDs. Consequently, the light‐emitting diodes based on the green QDs realize a maximum luminance of 15606 cd m^−2^, a peak external quantum efficiency of 10.6%, and a long half lifetime of > 5000 h.

## Graphical abstract

A quasi‐shell‐growth strategy for synthesizing highly luminescent green InP/ZnSe/ZnS quantum dots (QDs) with high photoluminescence quantum yields over 90% and narrow full‐width at half‐maximum of 36 nm is reported. The resulting QD light‐emitting diodes based on these core/shell QDs with improved optoelectronic properties realize a high brightness of 15606 cd m^−2^ and a long operational lifetime of over 5000 h.

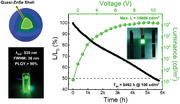

## 1 Introduction

Indium phosphide (InP) quantum dots (QDs) have been demonstrated as one of the most promising emitters for light‐emitting diodes (LEDs), benefiting from their emission wavelength tunability, good color purity, environmental friendliness, etc.^[^ ^1^, ^2^, ^3^, ^4^, ^5^, ^6^ ^]^ Despite great advancements achieved for InP QDs synthesis in recent years, the overall photoluminescence (PL) and electroluminescence (EL) performance of green InP QDs are still inferior compared to their red counterparts, due to more surface defects existing in small‐size QDs that lead to low PL (PLQYs) and inefficient charge recombination in device.^[^ ^7^, ^8^, ^9^, ^10^ ^]^ In addition, although less defects of InP cores with high crystallinity could be obtained at elevated temperatures, the Ostwald ripen often occurs that causes the non‐uniform isotropic growth of InP cores and thus degrades the size distribution of QDs, which is also unfavorable for their luminescence performance.^[^ ^11^ ^]^

Enormous efforts have been devoted to improving the quality of InP‐based QDs by researchers worldwide. Fluorides such as hydrofluoric acid (HF) were used to passivate the defects of InP cores by eliminating the undercoordinated surface dangling bonds and etch away the InPO_x_ oxidation layer prior to coating a shell, resulting in promoted QYs for InP QDs with more uniform morphology.^[^ ^12^, ^13^, ^14^, ^15^, ^16^, ^17^, ^18^ ^]^ However, the utilization of fluorides is dangerous and it is also difficult to control the amount of HF precisely, which often causes the reoxidation of InP core and installation of shallow hole traps.^[^ ^19^ ^]^ Moreover, due to the strong coordinating strength of In ligands, the reaction conditions need to be very harsh, easily leading to the uneven particle size distribution and broadened emission spectra of InP QDs caused by Oswald ripening.^[^ ^20^, ^21^ ^]^ Nowadays, it remains a great scientific challenge to prepare high‐quality core/shell structured InP QDs, especially for the QDs with shorter wavelengths due to their smaller particle sizes and often more defects on the core surface.

Here, we develop a quasi‐shell‐growth strategy (QS strategy) for synthesizing highly luminescent green InP/ZnSe/ZnS QDs with a high PL QY of over 90% and a narrow full‐width at half‐maximum (FWHM) of 36 nm. A quasi‐ZnSe shell (Q‐ZnSe) was formed by the adsorption of Zn and Se monomers at the beginning of InP nucleation, which plays multiple roles in suppressing the Oswald ripening of InP core under high temperatures to ensure the isotropic growth of InP QDs with improved size distribution, passivating the defects in In‐terminated surface of InP core, as well as avoiding the lattice strains at the core/shell interface of QDs caused by the direct coating shell of high‐temperature. As a result, the resulting QLED exhibits significantly enhanced luminous efficiency, high brightness, and long operational lifetime.

## 2 Results and Discussion

**Figure 1:**
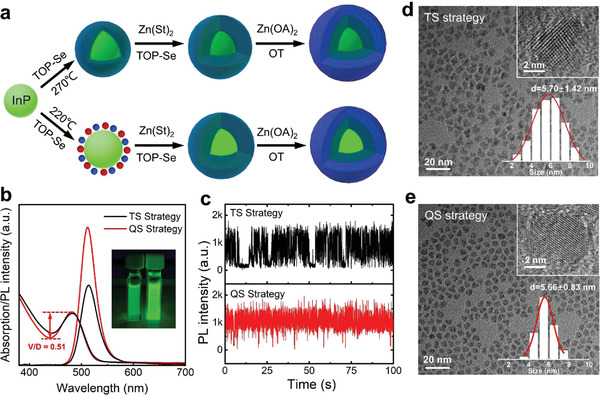
a) Synthetic schematic of InP/ZnSe/ZnS QDs with TS (up route) and QS (down route) strategy. b) Absorption and PL spectra of InP/ZnSe/ZnS QDs. Inset: the fluorescent image of QDs with TS (left) and QS (right) strategies under UV light irradiation. c) PL intensity traces of single dot with different strategies. d,e) TEM images of InP/ZnSe/ZnS QDs with the above two strategies. Insets show the HRTEM images of QDs.

The synthetic procedures for InP/ZnSe/ZnS QDs are illustrated in **Figure** **1**a, in which the down route represents the developed QS strategy, and the traditional synthesized strategy (TS strategy, the up route) is also included for comparison. Experiment details are provided in the Experimental Section. In a typical QS strategy, the Se precursors are injected at a lower temperature of 220 °C, namely, the initial nucleation stage of InP, to coat uniformly on InP cores to form the quasi‐ZnSe (Q‐ZnSe) shell rather than the bulk ZnSe shell directly. As increasing the temperature to 270 °C, the Q‐ZnSe shell is gradually transformed into the ZnSe shell. By contrast, the injection of Se precursors was used at the higher temperature of 270 °C for the direct formation of ZnSe shell in the TS strategy. As shown in Figure 1b, the QDs prepared by the QS strategy exhibit an increased absorption valley/depth (V/D) ratio of 0.51 and a narrower linewidth of 36 nm than those of QDs prepared by the TS strategy, indicating their more uniform size distribution. The corresponding PLQY is also enhanced from 75% for TS strategy‐assisted QDs to 91% for QS strategy‐assisted QDs. From the PL trail of a single QD in Figure 1c, the QS strategy assisted QDs exhibit the constant “on” state, considered as nearly non‐blinking behavior, which reveals the suppressed Auger recombination.^[^ ^22^, ^23^ ^]^ In addition, the TS strategy assisted QDs present nonuniform morphology with different shapes (Figure 1d), while the QS strategy assisted QDs display a regular spherical shape (Figure 1e), resulting from that the Q‐ZnSe shell can suppress the Ostwald ripening of InP core at high temperatures. Moreover, the average size of QDs is measured to be 5.70 ± 1.42 (TS‐strategy) and 5.66 ± 0.83 nm (QS‐strategy), respectively. The reduced size variation range for QS strategy assisted QDs also agree well with the increased absorption V/D ratio and the narrower FWHM in Figure 1b, further confirming their improved size distribution compared to the TS strategy assisted QDs. TEM images of InP core, InP/ZnSe, and InP/ZnSe/ZnS QDs prepared based on QS strategy are summarized in Figure S1, Supporting Information.

**Figure 2:**
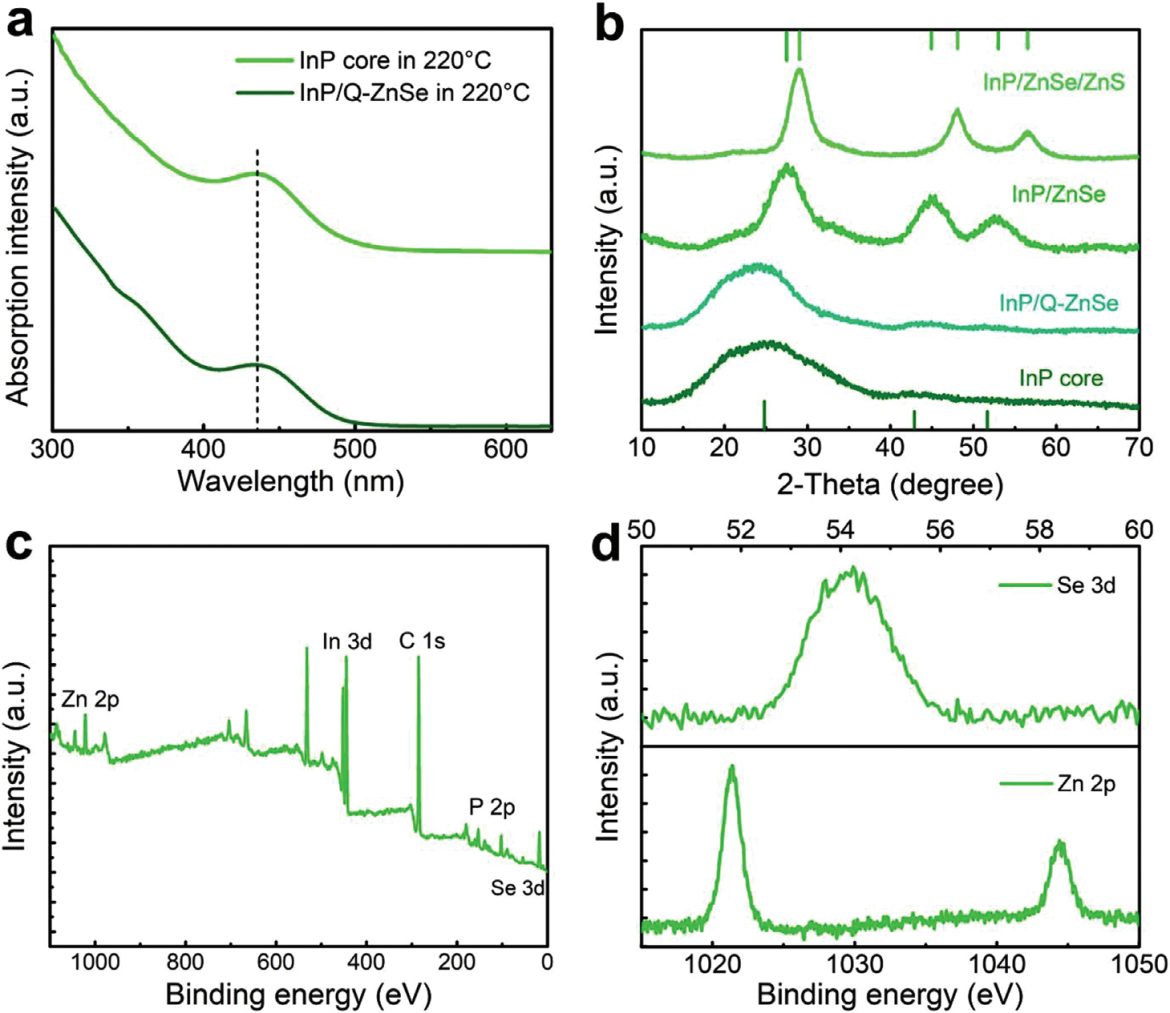
a) Absorption spectra of InP core and InP/Q‐ZnSe QDs at 220 °C. b) XRD patterns of InP, InP/Q‐ZnSe, InP/ZnSe, and InP/ZnSe/ZnS QDs. c) Wide‐scan XPS spectrum for InP/Q‐ZnSe QDs. d) High‐resolution XPS spectra of Se 3d and Zn 2p elements for InP/Q‐ZnSe QDs.

The formation of Q‐ZnSe shell plays a critical role in achieving high‐quality InP/ZnSe/ZnS core/shell QDs. Both the InP cores and InP/Q‐ZnSe QDs are sampled at 220 °C to investigate the growth dynamics of the Q‐ZnSe shells. As shown in **Figure** **2**a, the first absorption maximums (*λ*_ex_) for both the samples are observed at the same wavelength of 435.6 nm, excluding the formation of bulk ZnSe shell if the transfer of electrons from InP core to ZnSe shell would induce the red‐shift of *λ*_ex_.^[^ ^24^ ^]^ The X‐ray diffraction patterns (XRD) of InP core, InP/Q‐ZnSe, InP/ZnSe, and InP/ZnSe/ZnS QDs were performed and compared (Figure 2b) to verify the growth of Q‐ZnSe shell. Note that the three distinct peaks at (111), (220), and (311) of InP/Q‐ZnSe QDs are consistent with the phase of InP cores (JCPDS No. 73–1983). As increasing the temperature, the Zn and Se monomers are adsorbed on InP cores rather than forming a bulk ZnSe shell in InP/Q‐ZnSe QDs, and then the ZnSe phase (JCPDS No. 80‐0021) appeared, evidencing the existence of Q‐ZnSe. In order to avoid the inaccuracy of absorption spectra and XRD measurements for InP and InP/Q‐ZnSe QDs caused by the shedding of Zn and Se monomers, X‐ray photoelectron spectroscopy (XPS) spectra of purified InP/Q‐ZnSe QDs were also analyzed (Figure 2c,d). Besides the In (444.35 eV) and P (132.75 eV) peaks, the peaks for Zn 2p and Se 3d are observed at 1021.36 and 54.14 eV, respectively, which further confirms the formation of Q‐ZnSe shell on InP core.

**Figure 3:**
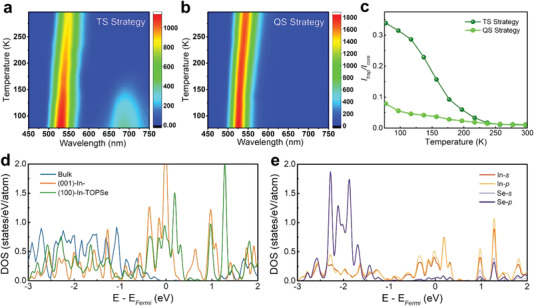
2D temperature‐dependent PL spectra for a) InP/ZnSe and b) InP/Q‐ZnSe QDs from 77 to 297 K, respectively. c) Temperature dependence of the relative PL intensities of trap emissions for InP/ZnSe and InP/Q‐ZnSe QDs. d) Electronic DOS for In atoms in bulk InP, and on the clean and TOP‐Se passivated In‐terminated (100) surface. e) Electronic DOS of Se and In for the In‐terminated (100) surface with adsorbed TOP‐Se.

Temperature‐dependent PL measurements of InP/Q‐ZnSe and InP/ZnSe QDs with the same amount of TOP‐Se (0.1 mmol) from 77 to 297 K were carried out to investigate the effect of Q‐ZnSe on InP core, as shown in **Figure** **3**a,b. For both samples, the red shift of PL peaks as well as the broadening of FWHMs are evidently observed as the increase in temperature, resulting from the band gap shrinkage of InP and the electron‐phonon coupling effect.^[^ ^25^, ^26^ ^]^ Notably, for TS strategy assisted QDs, there exist strong emission peaks at longer wavelengths region (650–750 nm),^[^ ^27^ ^]^ which originates from the trap states and disappear in QS strategy assisted QDs, suggesting that the defects of InP core have been effectively passivated by the growth of the Q‐ZnSe shell. For intuitive comparison, we plotted the corresponding PL intensities of these two emission peaks (Figure 3c). The lower trap emission intensities demonstrated fewer surface traps in QS strategy assisted QDs with the increase in temperature, which is also conducive to the growth of ZnSe and ZnS shells, thereby leading to high‐quality InP/ZnSe/ZnS QDs with PLQYs. Density functional theory (DFT) calculations were conducted to deeply analyze how the Q‐ZnSe passivate the defects of InP cores. According to the electronic density of states (DOS) in Figure 3d, for the In‐terminated (100) surface, there is a peak at ≈0.7 eV above the Fermi level, sitting in the middle of bandgap, which can be eliminated by the passivation of TOP‐Se. Figure 3e also demonstrates that In and Se orbitals have strong hybridization, indicating the existence of chemical bonding between them. Moreover, the calculated adsorption energy for TOP‐Se on In‐terminated and P‐terminated (100) surfaces are 1.0 and 0.7 eV, respectively, further suggesting that TOP‐Se is more likely to be adsorbed on In‐terminated surfaces^[^ ^28^ ^]^ for the formation of Q‐ZnSe shell (Figure S2, Supporting Information).

**Figure 4:**
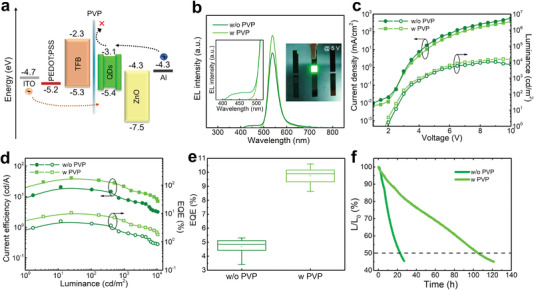
a) Energy level of InP‐based QLED. b) EL spectra of QLEDs with/without PVP interlayer. Inset shows an operational device with an emitting area of 4 mm^2^ c) *J‐L‐V* and d) CE‐EQE‐L characteristics for InP‐based QLEDs with/without PVP interlayer. e) Distribution of EQE values and f) the operating lifetime characteristics of InP‐based QLEDs with/without PVP interlayer.

To evaluate the electroluminescence performance of the synthesized InP/Q‐ZnSe/ZnS QDs, the green QLEDs with a standard device structure of ITO/PEDOT: PSS/TFB/QDs/ZnO/Al were first fabricated and measured (Figures S3–S5, Supporting Information). The devices based on QS strategy assisted InP QDs show a maximum luminance of 10 455 cd m^−2^ and a peak external quantum efficiency (EQE) of 5.3%, more than twofold improvement compared to the control QLED (max. luminance of 2976 cd m^−2^ and EQE of 2.5%). It is worth pointing out that there is a parasitic emission at ≈ 450 nm often existing in the EL spectra of green InP QLEDs owing to that the highly delocalized electrons would transport across the QDs layer and enter into the hole transport layer, thus degrading the device performance and color purity.^[^ ^8^ ^]^ Therefore, an ultrathin interlayer of polyvinyl pyrrolidone (PVP) was inserted between TFB and QDs in these devices to inhibit the transfer of electrons from QDs to TFB layer. The corresponding energy level diagram is displayed in **Figure** **4**a, where the conduction band minimum and valence band maximum of InP/ZnSe/ZnS QDs are measured to be 3.1 and 5.4 eV (Figure S6, Supporting Information), and the energy level of other functional layers were obtained from literature.^[^ ^29^, ^30^, ^31^, ^32^, ^33^, ^34^ ^]^ Figure 4b shows the EL spectra of QLEDs under a driving voltage of 5 V. Compared with the reference device without PVP interlayer, the QLEDs with the PVP interlayer exhibit a higher EL emission intensity, and the parasitic emission of TFB disappeared with the insertion of PVP interlayer, demonstrating the effective confinement of electrons into InP QDs.^[^ ^35^ ^]^ Figure 4c,d shows the current density‐luminance‐voltage (*J‐L‐V*) and current efficiency‐external quantum efficiency‐luminance (CE‐EQE‐L) characteristics of devices. The device with PVP interlayer exhibits a peak luminance of 15 606 cd m^−2^ with a peak EQE of 10.6%, much better than those of the control device with a peak luminance of 10 455 cd m^−2^ and a peak EQE of 5.3%. The detailed parameters of QLEDs are summarized in Table S1, Supporting Information. The enhancement of EL performance is attributed to the improved charge recombination in InP QDs layer, originating from the PVP‐induced improved confinement of electrons. Meanwhile, atomic force microscopy measurements were conducted to analyze the film morphology of QDs upon different layers (Figure S7, Supporting Information). The root mean square of QDs films is reduced from 0.962 to 0.631 nm after the insertion of PVP layer, which favors the device performance.^[^ ^20^, ^36^ ^]^ Figure 4e is the distribution of EQE values from 20 devices, showing an average peak EQE of 9.8% for QLEDs with PVP and 4.8% for control QLEDs, respectively, indicating the good reproducibility of our devices based on InP/ZnSe/ZnS QDs. Since the operational stability is important to QLEDs, the half‐lifetime comparison between the QLEDs with or without PVP interlayer was conducted after being encapsulated. As is shown in Figure 4f, the QLEDs with PVP interlayer show a better half‐lifetime of 105 h at an initial luminance of 1182 cd m^−2^, equivalent to 5462 h at the initial luminance of 100 cd m^−2^, which is over fivefolds longer than that of reference device (≈23 h @ 1056 cd m^−2^). The substantially improved device lifetime is mainly due to the improved PL properties of QS strategy assisted InP/ZnSe/ZnS QDs and the better charge recombination inside the device driven by PVP interlayer.

## 3 Conclusion

In summary, we developed a quasi‐shell growth strategy for high‐quality InP/ZnSe/ZnS core/shell QDs, during which the intermediate Q‐ZnSe shell plays a vital role in not only passivating the defect states of InP cores but also suppressing the Ostwald ripening process. The resulting InP/ZnSe/ZnS QDs present the highest PLQY of 91% and a uniform spherical morphology with a narrow size distribution. Further, the electroluminescence performance of the InP based QDs was also improved by introducing a very thin PVP interfacial layer that simultaneously improves the color purity, charge balance, and film surface quality and therefore, the resulting green QLEDs exhibit a high EQE of 10.6%, brightness of over 15 000 cd m^−2^, and a long operational lifetime of 5462 h. This work suggests an effective strategy as well as a deep understanding for achieving efficient Cd‐free QLEDs from both material and device perspectives.

## 4 Experimental Section

### Materials

Indium (III) acetate (99.999%), palmitic acid (98%, PA), zinc stearate (10% Zn basis), 1‐octadecene (90%, ODE), oleic acid (90%, OA), selenium powder (99.99%), sulfur powder (99.998%), PVP, Tetramethylammonium hydroxide (≥97%, TMAH), anhydrous zinc acetate (99.99%), and dimethyl sulfoxide (≥99.9%, DMSO) were purchased from Sigma‐Aldrich. Tris(trimethylsilyl)‐phosphine (98%, (TMS)_3_P) and trioctylphosphine (97%, TOP) were purchased from Strem Chemicals. Poly[(9,9‐dioctylfluorenyl‐2,7‐diyl)‐alt‐(4,4′‐(N‐(4‐butylphenyl) (TFB) was purchased from Xi'an Polymer Light Technology Corp. Acetone (extra dry, >99%), ethanol (extra dry, >99%), ethyl acetate (extra dry, >99%), and octane (extra dry, >99%) were purchased from Acros. All chemicals were used without further purification.

### Preparation of TOP‐Se (2M)

Selenium powder (10 mmol) was dissolved in 5 mL of TOP and stirred at room temperature for 12 h in a nitrogen glovebox.

### Preparation of TOP‐S (2M)

Sulfur powder (10 mmol) was dissolved in 5 mL of TOP and stirred at room temperature for 12 h in a nitrogen glovebox.

### Synthesis of InP/ZnSe/ZnS QDs

Indium acetate (0.2 mmol), palmitic acid (0.6 mmol), zinc stearate (0.3 mmol), and 10 mL of ODE were mixed in a 50 mL three‐neck flask. The mixture was evacuated (120 °C, 1h) and cooled to room temperature under N_2_ atmosphere. Subsequently, 0.1 mmol (TMS)_3_P mixed with 1 mL of TOP were injected quickly into the flask and then heated the flask (for TS strategy, the temperature was raised to 270 °C; for QS strategy, the temperature was raised to 220 °C, and 0.1 mmol TOP‐Se in 1 mL ODE was drop wised into the flask for 3 min before the temperature increased to 270 °C). As the temperature reaches 270 °C for 3min, 0.1 mmol TOP‐Se in 1mL ODE was injected and maintained at the same temperature for 10 min. Then 0.7 mmol zinc stearate in 6 mL ODE and 0. 5 mmol TOP‐Se in 5 mL ODE was injected slowly and maintained at the same temperature. After waiting for 1h, 2 mmol zinc stearate in 20 mL ODE and 2.2 mmol TOP‐S in 20 mL ODE were injected respectively into the flask at the same temperature for 2 h. The reaction lasts for another 30 min after the last injection. The mixture was cooled down to room temperature and the QDs were precipitated in air by adding 25 mL of acetone to 5 mL of the crude solution in air and centrifugated at 7800 rpm for 3 min. Finally, the QDs were re‐dispersed in octane and stored in an N_2_‐filled glovebox.

### Synthesis of ZnO Nanoparticles

ZnO nanoparticles were synthesized with the mixture of TMAH (5.5 mmol) dissolved in ethanol solution (10 mL) and anhydrous zinc acetate (3 mmol) dissolved in DMSO solution (30 mL). After stirring for 2 h under room temperature, the ZnO nanoparticles were washed with excess ethyl acetate twice and re‐dispersed in ethanol.

### Device Fabrication

The ITO‐coated glass substrates were cleaned consecutively with detergent, acetone, and isopropanol under sonication for 40 min each, and were then treated with O_3_‐plasma for 10 min. Subsequently, a PEDOT:PSS layer was spin‐coated onto ITO substrates at 4000 rpm for 40 s, followed by annealing at 155 ℃ for 20 min. Then, the coated substrates were transferred into an N_2_‐filled glove box for spin coating TFB, PVP, QDs, and ZnO layers. The TFB layer was spin coated at 2000 rpm for 50 s using an 8 mg mL^−1^ chlorobenzene, followed by baking at 150 °C for 20 min. The PVP solution (0.3 wt.% in ethanol) was spin‐coated at 5000 rpm for 50 s, followed by baking at 80 °C for 10 min. The QDs and ZnO nanoparticles were prepared by spin coating at 2000 rpm for 50 s and baked at 90 °C for 10 min. Finally, the Al electrode (110 nm) was deposited by thermal evaporation under a based vacuum of ≈2.5 × 10^−4^ Pa.

### DFT Simulations

Density functional calculations were performed by using the Vienna Ab initio Simulations Package (VASP) and projected augmented wave (PAW).^[^ ^37^ ^]^ The exchange‐correlation interaction was treated with the generalized gradient approximation (GGA) in the Perdew, Burke, and Ernzerhof (PBE) parameterization.^[^ ^38^ ^]^ The zinc blende InP structure was fully relaxed and the energy was converged within 10^−6^ eV cell^−1^, with the cutoff energy of plane‐wave basis set as 400 eV and a Gamma‐centered k‐point set of 6×6×6. TOP‐Se was modeled with (CH3)_3_PSe and fully relaxed in a cubic supercell with more than 10 Å spacing. The same cutoff energy and similar dense k‐point set were used for slab calculations, except that only one k‐point was used for the direction normal to the surface. A vacuum spacing no less than 15 Å was used in all slab calculations.

### Characterizations

The PL spectra, PLQY, and PL lifetime were measured using an Edinburgh FLS920 PL spectrometer. Absorption spectra were measured using a PerkinElmer Lambda 950 UV–vis‐NIR spectrometer. Single‐dot PL spectroscopy was performed with a Zeiss Elyra P.1 super‐resolution microscope. The evaporation rates and thicknesses of Al were monitored in situ by a quartz crystal microbalance. XRD patterns were obtained using a Bruker D8 Advance diffractometer with Cu K*α* radiation range from 10° to 70° at a scanning rate of 6° min^−1^. The transmission electron microscopy images of the QDs were made on Talos F200X/TALOS F200X. The XPS and UPS measurements were performed by using a Thermo Scientific Escalab 250Xi. He (I) ultraviolet radiation source (21.22 eV) from a He discharge lamp was used in UPS measurements. The cross‐sectional TEM image of the QLED was characterized by the dual‐beam focused ion beam which was performed with a scanning electron microscope and Omniprobe AutoProbe 200.2 robot hand. The thickness of PVP film was determined by step profiler (D‐500 stylus Profilometer). The temperature‐dependent PL spectra were measured through a Newton CCD (model no. DU920P‐BU) integrated with Shamrock spectrometer (model no. SR‐750‐D1‐R) with a laser excitation (442 nm) at different temperatures in a helium cryostat (CRYO Cool‐G2B‐LT). The *J‐L‐V* and CE‐EQE‐L characteristics were measured by Keithley 2400 source meter and PR‐670 Spectra Colorimeter. The half‐lifetime of QLEDs was tested through ZJZCL‐1 OLED aging lifespan test instrument.

### Statistical Analysis

Microsoft Excel and OriginPro (version 2016,Originlab Corp.) were used for the statistical analysis of the data presented in this work. Calculation method was described in DFT Simulations. Data presentation and sample size were exhibited as the mean ± standard deviation (SD).

## Conflict of Interest

The authors declare no conflict of interest.

## Supporting information

Supporting Information

## Data Availability

The data that support the findings of this study are available from the corresponding author upon reasonable request.
