# Supplementary material for: Quasi‐Shell‐Growth Strategy Achieves Stable and Efficient Green InP Quantum Dot Light‐Emitting Diodes
Source: Adv Sci (Weinh). 2022 May 26;9(21):2200959. doi: 10.1002/advs.202200959 (PMC9313472; doi:10.1002/advs.202200959)
Supplement: Supplementary file 1 — Supporting Information [file ADVS-9-2200959-s001.pdf]

ADVANCED SCIENCE
Open Access

# Supporting Information

for *Adv. Sci.*, DOI 10.1002/advs.202200959

Quasi-Shell-Growth Strategy Achieves Stable and Efficient Green InP Quantum Dot Light-Emitting Diodes

*Qianqian Wu, Fan Cao, Sheng Wang, Yimin Wang, Zhongjiang Sun, Jingwen Feng, Yang Liu, Lin Wang, Qiang Cao, Yunguo Li, Bin Wei, Wai-Yeung Wong and Xuyong Yang**

*Supporting Information*

**Quasi-Shell-Growth Strategy Achieves Stable and Efficient Green InP Quantum Dot Light-Emitting Diodes**

*Qianqian Wu, Fan Cao, Sheng Wang, Yimin Wang, Zhongjiang Sun, Jingwen Feng, Yang Liu, Lin Wang, Qiang Cao, Yunguo Li, Bin Wei, Wai-Yeung Wong and Xuyong Yang**

Q. Wu, F. Cao, Dr. S. Wang, Y. Wang, Z. Sun, Dr. L. Wang, Prof. B. Wei, Prof. X. Yang
Key Laboratory of Advanced Display and System Applications of Ministry of Education, Shanghai University,
149 Yanchang Road, Shanghai 200072, P. R. China
E-mail: yangxy@shu.edu.cn

J. Feng, Dr. Y. Liu
BOE Technology Group Co., Ltd., Beijing 100176, P. R. China

Q. Cao
The Institute of Technological Sciences, Wuhan University, Wuhan 430072, P. R. China

Prof. Y. Li
CAS Key Laboratory of Crust-Mantle Materials and Environments, School of Earth and Space Sciences, University of Science and Technology of China, Hefei 230026, P. R. China

Prof. W.-Y. Wong
Department of Applied Biology and Chemical Technology, The Hong Kong Polytechnic University, Hung Hom, Kowloon, Hong Kong, P. R. China

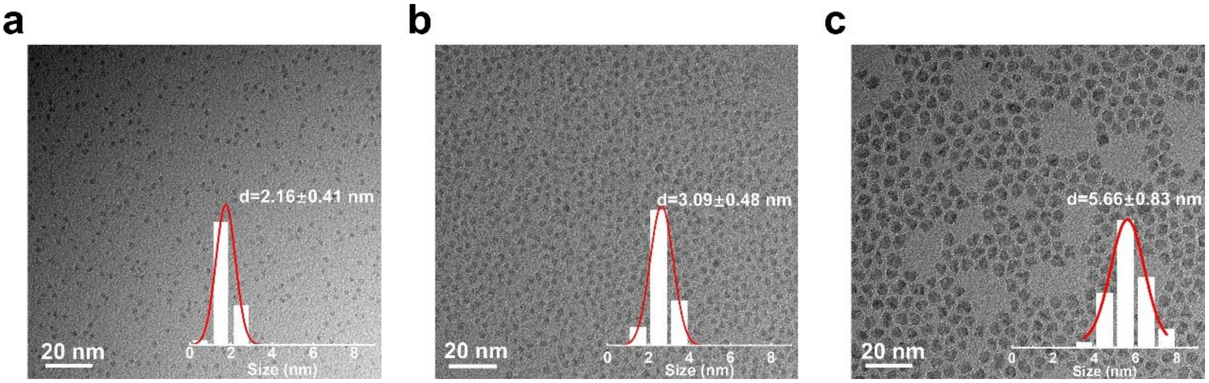


**Figure S1.** TEM images and their corresponding size distributions of (a) InP cores, (b) InP/ZnSe, and (c) InP/ZnSe/ZnS QDs. Red lines indicate Gaussian fitting of the size distribution.

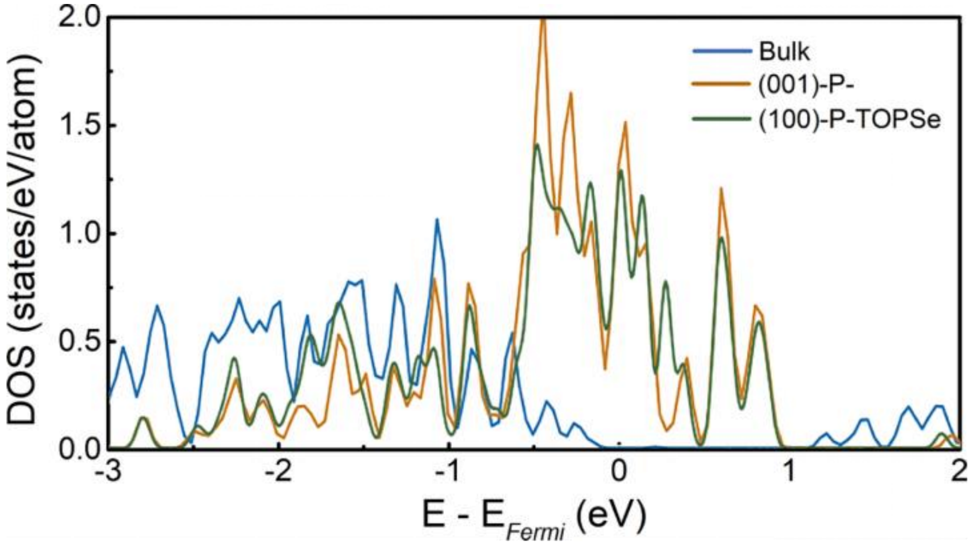


**Figure S2.** Electronic density of states for P atoms in bulk InP, and on the clean and TOP-Se passivated P-terminated (100) surface.

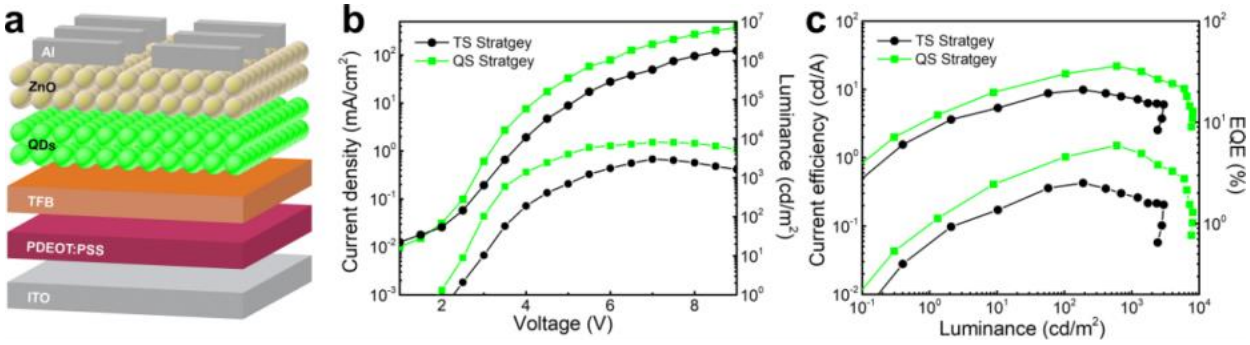


**Figure S3.** (a) Schematic device structure of InP-based QLED. (b) *J-L-V* and (c) *CE-EQE-L* characteristics for QLEDs based on TS or QS strategy.

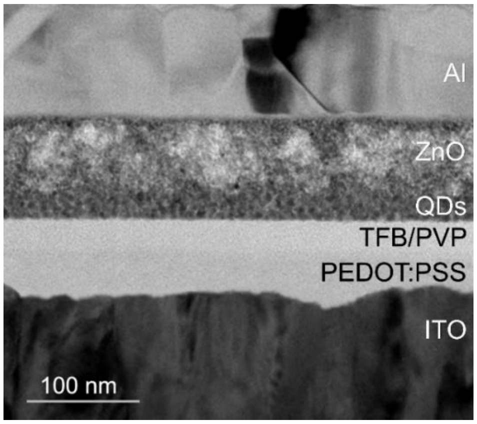


**Figure S4.** Cross-sectional TEM image of the QLED.

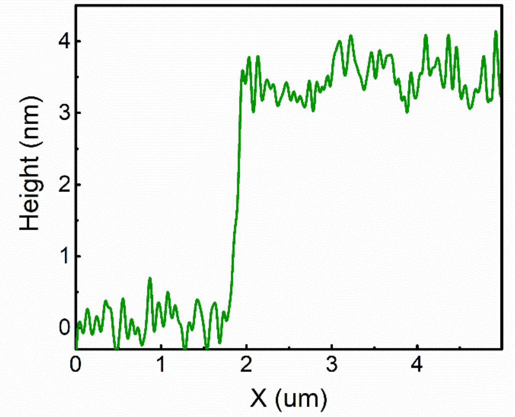


**Figure S5.** Thickness measurement of PVP thin layer.

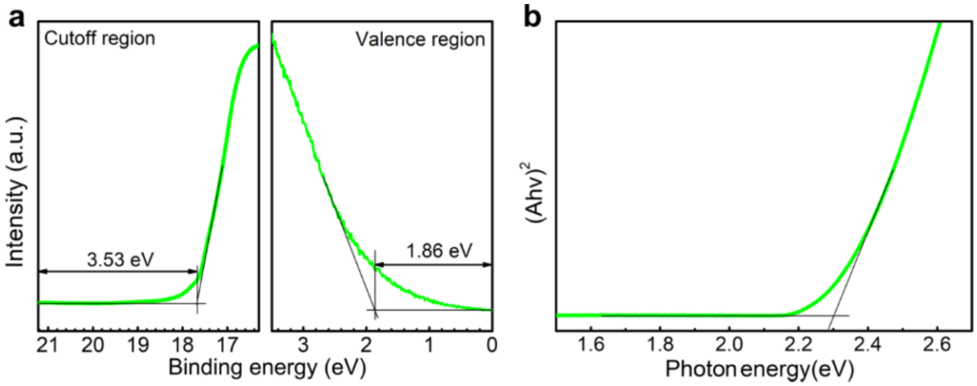


**Figure S6.** (a) UPS spectra of the secondary-electron cut-off region and the valence band edge region of InP/ZnSe/ZnS film. (b) Dependence of $(Ahv)^2$ of InP/ZnSe/ZnS film upon the incident photon energy (hv).

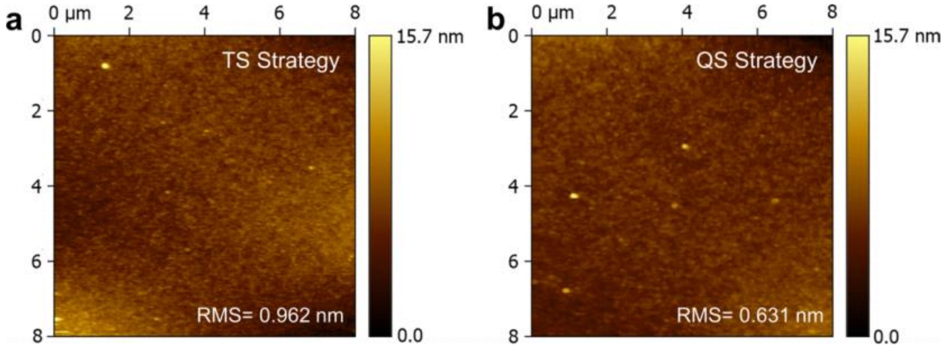


**Figure S7.** AFM images of InP/ZnSe/ZnS QD films upon TFB and TFB/PVP layers.

**Table S1.** Summary of the detailed output parameters for the devices.

| Devices | $V_{on}$ (V) | Max. L ($cd/m^2$) | Max. CE (cd/A) | Max. EQE (%) |
|---|---|---|---|---|
| TS strategy | 2.3 | 2976 | 9.8 | 2.5 |
| QS strategy | 2.0 | 10455 | 20.5 | 5.3 |
| QS strategy & with PVP | 1.8 | 15606 | 40.7 | 10.6 |
